# Brillouin Scattering and First-Principles Studies of BaMO_3_ (M = Ti, Zr, and Cu) Perovskites

**DOI:** 10.3390/ma15196747

**Published:** 2022-09-28

**Authors:** Md Al Helal, Seiji Kojima

**Affiliations:** 1 Department of Physics, Begum Rokeya University, Rangpur 5400, Bangladesh; 2 Graduate School of Pure and Applied Sciences, University of Tsukuba, Ibaraki 305-8573, Japan

**Keywords:** Brillouin scattering, first-principles calculation, perovskite, barium oxide, ferroelectric

## Abstract

Perovskite oxides with the general formula ABO_3_ comprise a large number of families among the structures of oxide-based materials, and currently, several perovskite structures have been identified. From a variety of compositions and structures, various functions are observed in perovskite compounds, and therefore, they became very useful for various applications in the electronic and medical industries. One of the most puzzling issues for perovskite compounds is the understanding of the vibration and relaxation dynamics in the gigahertz range. In that sense, the micro-Brillouin scattering system is a very effective tool to probe the gigahertz dynamics, and also, first-principles calculations can be used to describe the phonon structure with different atomic contributions. The micro-Brillouin scattering system and first-principles calculations provide the fundamental information on a variety of vibration and relaxation processes related to structural phase transitions under different external conditions such as temperature, electric field, and pressure. This review article summarizes the Brillouin scattering and first-principles studies on BaMO_3_ (M = Ti, Zr, and Cu). Through a detailed analysis of the existing results, we summarize the existing limitations and future perspectives in these research areas, which may propel the development of different perovskite ferroelectrics and extend their practical application areas.

## 1. Introduction

In solid-state physics, crystals with the ABO_3_-type (A: Large cation with different valence state and B: Transition metal) perovskite family structure are being widely studied because they put up most of the metal ions in the periodic table owing to its substantial number of different ions [1]. These types of oxides are significant in the field of various technological applications in modern electronics, non-linear optics, catalysis, refractories, geophysics, particle accelerators, etc. [2,3,4,5,6]. One of the major benefits of perovskite-structured oxides is that they can accept considerable substitutions in one or both cationic sites (A and B sites) while retaining their original crystal structures [7]. Currently, these types of compounds have several outstanding applications in the electronic industries such as piezoelectric devices, transducers, random access memories, tunable microwave device displays, sensors, capacitors, and wireless communications [8,9,10,11,12].

**Figure 1:**
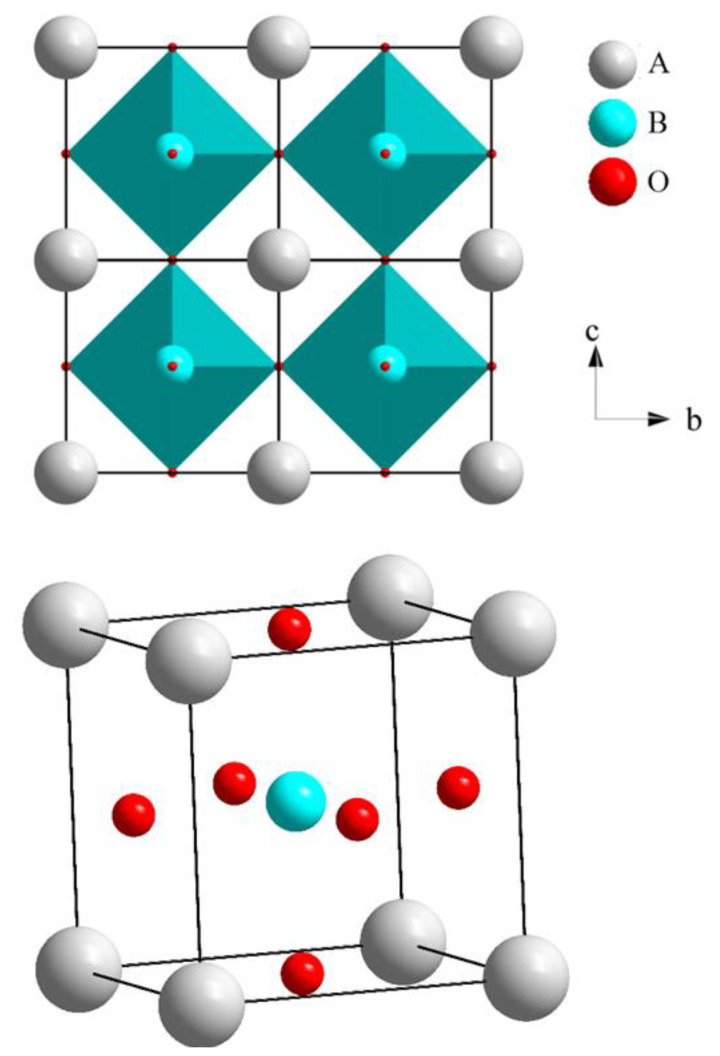
The 2D and 3D view of ABO_3_-type cubic perovskites.

The ideal structure of perovskite is cubic, which is shown in Figure 1. Although few compounds have this pure cubic phase, many perovskites have a slightly distorted phase with lower symmetry (e.g., tetragonal, orthorhombic, or rhombohedral). The lattice distortion in a crystal results from the off-center and/or displacement of the A or B site cations, which cause a ferroelectric phase transition owing to the polar phonon mode softening at a *Γ* point of the Brillouin zone. Meanwhile, the softening of the phonon mode can also appear due to the rotation of the oxygen octahedron at the *R* or *M* point of the Brillouin zone boundary [13]. Furthermore, many oxygen- or cation-deficient structures have been observed. The different categories of distortion in the perovskite materials are significantly related to their physical properties, in particular their ferromagnetic or ferroelectric properties.

Since its discovery, barium titanate (BaTiO_3_ (BTO)) has been studied extensively due to its remarkable ferroelectric properties, and it has been the subject of many studies for more than 50 years. Barium zirconate (BaZrO_3_ (BZO)) has attracted much attention from the scientific community due to its possible applications in hydrogen sensors and fuel cells. Barium cuprate (BaCuO_3_ (BCO)) is another promising perovskite material, which can be used to improve catalytic activity. The process of phase transition is different in these three crystals.

BTO is stable in a paraelectric cubic phase (space group *Pm*$\bar{3}$*m*) at high temperatures with five atoms per unit cell. Upon cooling, BTO exhibits three successive phase transitions from the paraelectric cubic phase to ferroelectric tetragonal (space group *P4mm*), orthorhombic (space group *Amm2*), and rhombohedral (*R3m*) phases at 393, 278, and 183 K, respectively. There exist numerous experimental and theoretical studies explaining the ferroelectric behavior of BTO single crystals. In spite of numerous reports, still, the true nature of the ferroelectric phase transition in BTO is controversial. The ferroelectricity and concomitant cubic to tetragonal distortion were initially discussed on the basis of displacive-type phase transition from the condensation of the soft optic mode [14,15]. However, with the lowering of temperature, the subsequent phase transitions of the orthorhombic and rhombohedral phases are inconsistent with the displacive nature [16,17]. This anomaly together with the results of low-temperature diffuse X-ray scattering [18] results in the development of a phenomenological order–disorder model [19]. In addition, the deviation of the refractive index from high-temperature linear behavior [20], first-order Raman spectra [21], the Curie-Weiss law for the permittivity [22], and significant softening of the longitudinal acoustic phonon [23,24] in the paraelectric cubic phase were observed in BTO crystals, which were ascribed to the existence of local polar clusters. Although BTO single crystals were studied more than 50 years, there is a significant lack of some decisive properties concerning the existence of the local polar cluster and the effect of external stress on this cluster, which must be attained to unlock the full potential of BTO for possible future technological applications.

The lattice dynamics and structural stability of BZO are found to be more complex, which has drawn great attention owing to its low-temperature structure and properties. As other perovskites, so far, no phase transition has been noticed in BZO at ambient pressure at any temperature. BZO has a cubic structure up to 2K according to neutron and X-ray diffraction studies [25]. However, these structural data disagree with the first-principles calculations [26,27], where the instability of the R or M phonon mode is observed due to the rotations of oxygen octahedra. In addition, a new transverse optic mode at about 2 THz was observed by the terahertz time-domain spectroscopy technique, which was forbidden in the consideration of cubic symmetry according to the infrared (IR) selection rule [28]. The origin of this peak was attributed to an IR active mode of the *P*$\bar{1}$ symmetry caused by local distortion. In BZO crystals, the first-order Raman active modes are observed, which is unfavorable for the cubic *Pm*$\bar{3}$*m* symmetry, indicating the lowering of the cubic symmetry [29,30]. However, the observed Raman spectra in BZO are consistent with the local tetragonal and orthorhombic symmetry [30,31,32].

One of the most interesting properties of perovskite materials is superconductivity. The discovery of superconductivity in La-Ba-Cu-O perovskite oxide with the K_2_NiF_4_-type layered structure containing the CuO_2_ plane was observed by Bednorz and Müller in 1984 [33]. After their report, significant attention has been paid to new types of high-temperature superconductors, mainly Cu-based perovskite oxides due to their excellent physical properties. It has been found that the presence of Cu on the B site cation is needed for superconductivity to occur in such perovskites. Due to the tremendous effort, a high value of transition temperatures (*T*_c_) was found in the YBa_2_Cu_3_O_7_ [34] and Bi_2_Sr_2_Ca_2_Cu_3_O_10_ [35] systems in 1987 and 1988, respectively, and currently, *T*_c_ has been further increased to 130–155 K in the HgBa_2_Ca_2_Cu_3_O_8+δ_ system [36]. So far, all the high-temperature superconductors are Cu-based oxides, and *T*_c_ is clearly associated with the Cu-O chain. In addition to superconductivity, Cu-based perovskites exhibit high electrical conductivity and catalytic activity, which are close to those of metals such as Cu. Very recently, another typical Cu-based superconducting BaCuO_3_ (BCO) perovskite has been reported [37,38], and *T*_c_ was found to be around 10 k.

In conventional dielectric spectroscopy measurement, it is very difficult to measure the broadband spectra between 1 and 1000 GHz. However, Brillouin scattering techniques enable the acquisition of such broad spectra by a single scan. To observe the dynamics of local polarization fluctuation in perovskite-type ferroelectrics, Brillouin scattering has been used as a very significant tool due to its sensitivity to local symmetry breaking related to the heterogeneity among a few methods, which can be used to directly identify the local structure. Another advantage of Brillouin scattering is that the spatial resolution is comparable to the wavelength of an excited laser, and it is possible to measure the acoustic phonon and optic soft mode in different types of perovskites.

In several applications, perovskites have been proven to be of great interest because of their meaningful physical properties for different device applications. In the present paper, we review the recent systematic studies on BaMO_3_ (M = Ti, Zr, and Cu) perovskites and discuss the existing limitations and future perspectives and research in these areas.

## 2. Methodology

### 2.1. Experimental Section

In solid-state physics, the determination of the velocity of an acoustic wave is given by the elastic constant and density. There are many methods to determine the elastic constants. Among them, the ultrasonic pulse-echo method and the mechanical resonance method have been well established [39,40]. However, as a non-contact and non-destructive method, Brillouin scattering spectroscopy is a powerful tool to measure the elastic constants with different external conditions, for example pressure, temperature, and electric field. Such a spectroscopic technique covers a large frequency range between 1 and 1000 GHz, and recently, it has been used to study not only the acoustic phonons, but also the optical soft mode [41], magnetic excitation [42], and localized excitation, such as fracton [43].

The frequency shift $\upsilon_{B}$ is proportional to the phase velocity V of the sound wave, which is measured by the following equation:

(1) $$\Delta \upsilon_{B}=\pm \frac{2n_{m}V}{\lambda_{0}}\sin\left(\frac{\theta }{2}\right)$$

where $\lambda_{0}$, $n_{m},$ and θ are the wavelength of incident light, the refractive index of the material to be studied, and the scattering angle, respectively.

**Figure 2:**
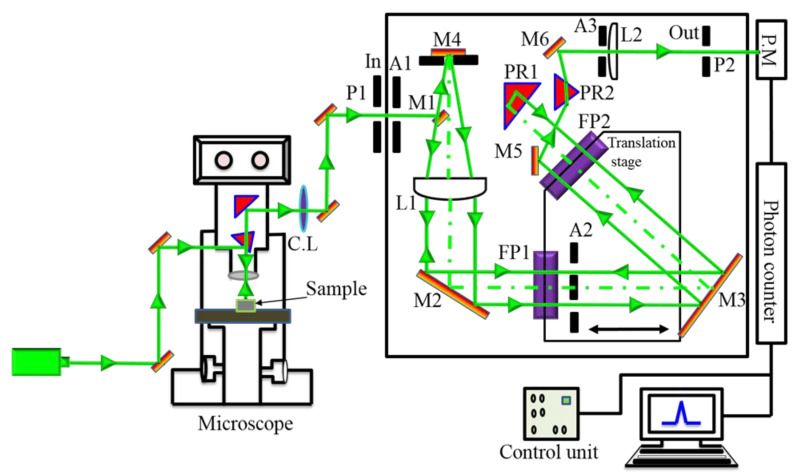
A schematic illustration of micro-Brillouin scattering setup.

The optical arrangement of a micro-Brillouin scattering apparatus is shown in Figure 2 [44]. The tandem multi-pass Fabray-Pérot interferometer, (FP1 and FP2), was designed by Sandercock (JRS, Zwillikon, Switzerland). The laser is focused on the sample with the help of a small mirror in the optical microscope arrangement. The scattered light enters FP1 via pinhole P1 and aperture A1 and is collected by the objective lens. The laser light is then directed via mirror M3 to FP2. The light strikes the 90°-oriented prism PR1 after the transmission through FP2, then is reflected backwards and returns to FP2. It continues via aperture A2 to FP1. The light is then passed via L1 and is focused onto mirror M4 with mirror M1 underneath, after the transmission through FP1. Mirror M4 then returns the laser light back to lens L1, and the light is projected to FP1 through M2 after collimation. The light is directed to PR2 through M5 after the final pass through FP2. A bandpass filter is formed by the combination of lens L2, prism PR2, and output pinhole P2. The light is projected onto the photomultiplier (PM) from mirror M6 via aperture A3, lens L2, and output pinhole P2. Finally, a conventional photon counter records the scattered signal.

To measure the micro-Brillouin scattering spectra, a 3 + 3 pass Sandercock-type tandem Fabray–Pérot interferometer (JRS TEP-1, JRS, Zwillikon, Switzerland), was used with a photon counting system. All the Brillouin scattering spectra were collected in a backwards scattering geometry [44,45]. The objective lens of the microscope focuses an incident laser beam with a wavelength of 532 nm and a power of about 100 mW onto the samples, and simultaneously, the scattered light is collected. The mirror space was set at 2 mm with a free spectral range of 75 GHz. The temperature of the sample was controlled by a heating and/or a cooling stage (THMS600, Linkam, Salfords, UK) with a stability of ±0.1 K. Two surfaces of the sample were coated with silver paste, and gold contact wires were attached to the electrodes to apply the electric field.

### 2.2. Theoretical Section

The density functional theory (DFT) formalism is the basis of first-principles calculations upon which the different properties of a material can be derived. Using DFT, without solving the multibody Schrödinger equation, the problem can be described with an equivalent non-interacting problem. The ground state structure is obtained after solving the Kohn–Sham equations [46]. The ultrasoft pseudopotential, given by Vanderbilt, was used to optimize the geometry, because it allows for a lower cut-off energy, without sacrificing transferability too much [47]. The ultrasoft pseudopotential method performs the calculations of the material by considering the electron density in the valance as the soft part and the core region as the hard part, where the cut-off energy is dramatically reduced and the computational efficiency increased appreciably. The bulk modulus, independent elastic constants, and shear modulus were estimated using the stress–strain method contained in the Cambridge Serial Total Energy Package (CASTEP) code. On the other hand, the norm-conserving pseudopotential was used for the phonon calculations. The system geometry was optimized under the energy convergence at a total energy of $0.5\;\times \;10^{-5}$ eV/atom, the maximum interatomic force 0.01 eV/Å, the maximum stress 0.02 GPa, and an ionic displacement of ${5\;\times \;10}^{-4}$ Å. Understanding the energy-dependent optical parameters is very crucial to explore the potential application of a new compound. The optical properties are very important from the viewpoint of a possible optoelectronic device applications. The optical constants’ spectra were extracted from the frequency-dependent dielectric function, ε(ω) = ε_1_(ω) + iε_2_(ω). The real part of the dielectric constant, ε_1_(ω), is calculated from the imaginary part, ε_2_(ω), using Kramers-Kronig transformations. The imaginary dielectric function, ε_2_(ω), can be calculated through the following equation:

(2) $$\varepsilon_{2}(\omega )=\frac{2e^{2}\pi }{{\Omega \varepsilon }_{0}}\underset{k,v,c}{\sum }{k\Psi }_{k}^{c}\left|û.ȓ|\Psi_{k}^{v}\right.{|}^{2}\delta \left(E_{k}^{c}\right.-E_{k}^{v}-\left.E\right)$$

Here, ȓ is the electron radius vector, û is the polarization vector of the incident electric field, *e* is the charge of the electron, ω is the frequency of light, and $\Psi_{k}^{c}$ and $\Psi_{k}^{v}$ are the conduction and valence band wave functions at k, respectively.

## 3. Typical Phenomena in BaMO_3_ (M = Ti, Zr, and Cu) Perovskites

### 3.1. Precursor Dynamics and Domain Switching in BaTiO_3_ Single Crystal

**Figure 3:**
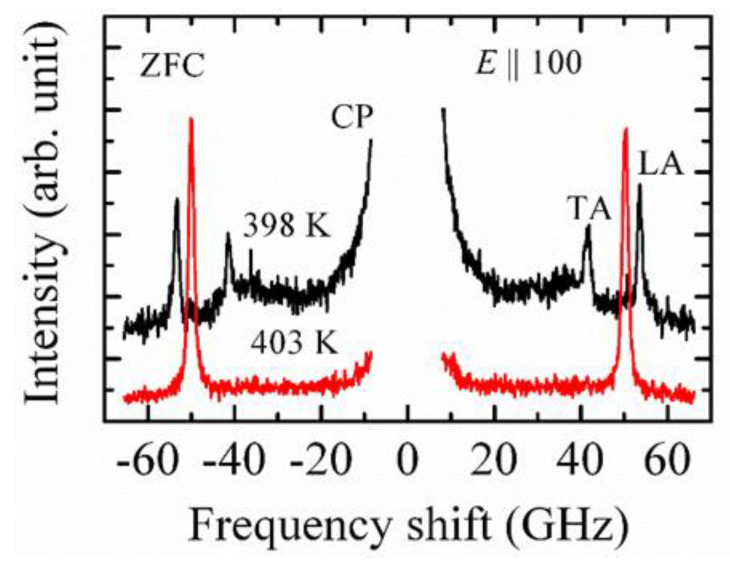
Brillouin scattering spectra at 398 and 403 K of (100)-oriented BaTiO_3_ single crystals.

BaTiO_3_ (BTO) is the first produced and most studied perovskite material with its superior dielectric, ferroelectric, and piezoelectric properties [48]. The high dielectric constants of BTO results from its crystal structure. In BTO crystals, three phonon frequencies were obtained from the FTIR measurements [49], located at around 40, 180, and 482 cm^−1^. The lowest frequency phonon mode is called the soft mode and is overdamped in a paraelectric phase. From the measured frequency, Luspin et al. [49] estimated the dielectric constant using the Lyddane-Sachs-Teller (LST) relation. Due to the soft mode dynamics, the dielectric constant increases markedly in the vicinity of the phase transition temperature upon cooling. The obtained dielectric constant gives a diverging value by approaching the Curie temperature, while the soft mode frequency remains nearly constant. To explain this discrepancy, two possible origins can be invoked. The first one comes from the contribution of impurities, which gives rise to the rapid increase of the dielectric constant due to the coupling with the soft mode. Another possibility is the existence of an additional relaxational mechanism in the microwave region. However, they had no direct conclusion about this point. Therefore, it is necessary to observe this relaxational mechanism by considering another approach. In BTO crystals, the size of the Ba ion is too large compared with the Ti ion, which is quite small and remains stable in the octahedral position. The Ti ion having a charge of +4 has the minimum energy position outside the center, in the direction of each of the O_6_ ions surrounding it. Due to the off-centering position of the Ti ions, the mechanism of the phase transition, in particular, the order-disorder- and/or displacive-type, is still under discussion. It was shown by Takahasi [50] and Comes et al. [18] that Ti ions go to the off-center position along eight equivalent [111] directions inside the cubic matrix with a non-zero local dipole moment. Therefore, this leads to a local polar nanoregion (PNR) in a cubic phase, which appears a few hundred degrees above the cubic to tetragonal phase transition temperature (*T*_c_), called the Burns temperature (*T*_B_). It is believed that PNRs play an important role in the appearance of the outstanding piezoelectric performance in electrical devices. In order to identify such types of nano-metric local polar regions, a low-frequency inelastic light scattering spectrum can be a very effective tool, because it can be used to directly probe the local symmetry and structure. Figure 3 shows a group of Brillouin scattering spectra at 398 and 403 K without any applied electric fields. 

It is pertinent to note that a longitudinal acoustic (LA) mode and a weak central peak (CP) appear in the Brillouin scattering spectrum above *T*_c_ (402 K). It is now believed that the CP appears in low-frequency light scattering spectra owing to the appearance of random fields, which originate from the displacement of Ti ions inside the oxygen octahedra [23,51]. It was also suggested from different experiments [52,53] that the motions of off-center Ti ions are correlated and create PNRs in which all the Ti ions are oriented in the same direction and fluctuate together. Above *T*_c_, previous theoretical reports also supported the creation of PNRs with local symmetry breaking [54,55]. Therefore, the presence of the CP in BTO crystals can be attributed to the polarization fluctuation of PNRs, which has also been observed in BTO-based ferroelectrics. However, to make further conclusions, it is very important to measure the broad CP by means of different experimental techniques. Ko et al. [56] measured the temperature dependence of the broad CP and discussed the critical slowing down behavior of the CP. They explained their result by the existence of polar clusters and the growth of these clusters with longer correlation lengths a few ten degrees above *T*_c_. To make still further conclusions, besides the temperature dependence, the electric field dependence of the broad CP is very important, which can reveal some unanswered questions. So far, there is no available result on the electric field dependence of the broad CP. Therefore, future experiments will test the growth of PNRs by measuring the broad CP by applying an electric field in different directions.

**Figure 4:**
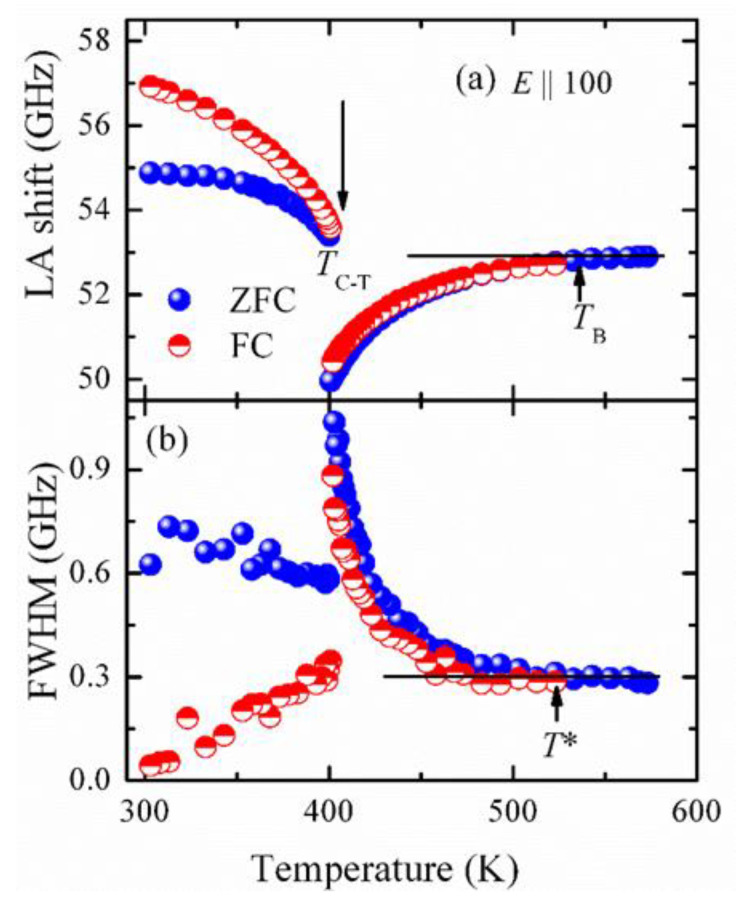
The temperature dependences of (**a**) frequency shift (υ_LA_) and (**b**) FWHM (ΓLA) of LA phonon mode of 100-BT single crystals measured in ZFC (blue solid symbols) and FC (red solid half-filled symbols) processes [24].

Another approach to the effect of PNRs is to observe the behavior of LA phonons in the zero field cooling (ZFC) and field cooling (FC) process. At first, the Brillouin spectra are measured without applying the electric field, called the ZFC process. To apply the electric field, the sample is heated above *T*_B_ and short circuited to remove any kind of memory effects. Then, an electric field of 1 kV/cm at a high temperature is applied, and the Brillouin spectra upon cooling are collected, called the FC process. After measuring the Brillouin scattering spectra in ZFC and FC, they are fit by a Lorentzian function convoluted by a Gaussian instrumental function from which the LA frequency shift ($\vartheta_{LA}$) and the FWHM ($\Gamma_{\mathrm{LA}}$) can be obtained. Figure 4a and b shows the $\vartheta_{LA}$ and $\Gamma_{LA}$ in the ZFC and FC processes, respectively. A sudden change of both $\vartheta_{LA}$ and $\Gamma_{LA}$ at around 402 K reflects the transition from the cubic to tetragonal phase.

Here, the interesting feature is that the LA frequency shift begins to deviate from its linear temperature dependence at around 533 K. It has been reported that the local strain fluctuation is created by the local polarization fluctuation inside a PNR. The macroscopic elastic anomaly and the piezoelectric coupling in a PNR occur due to the coupling between the local strain fluctuation in a PNR and acoustic phonons [57]. Therefore, we assigned this temperature as the Burns temperature, *T*_B_, where the dynamic PNRs begin to appear. On the other hand, the sound attenuation, related to $\Gamma_{LA}$, begins to increase abruptly towards the transition temperature at around 503 K, where the permanent PNRs arise, and the temperature is known as the intermediate temperature, T*. The observed *T*_B_ and T* in this study were somewhat higher than previously found by the Brillouin scattering results [56] carried out on BTO single crystals. The discrepancies of these results may appear because of the quality of the sample, because the authors noticed *T*_c_ at 373 K, which is far behind the actual *T*_c_ in BTO crystals. Interestingly, these two temperatures were also measured by the acoustic emission measurement [58] carried out on a single crystal of BTO, and the reported values of *T*_B_ were approximately between 530–570 K, while T^*^ was 506 K, which is in good agreement with the present Brillouin scattering results. Very recently, *T*_B_ was also measured by the resonant ultrasound spectroscopy (RUS) technique [59]. The onset of the frequency vs. temperature curve in the RUS method shows the deviation from the exponential softening towards a weaker temperature dependence near 560 K, which they denoted as *T*_B_. These two characteristic temperatures have been noticed in many relaxor ferroelectrics [18,60]. However, so far, this temperature has not been clearly identified [58].

The electric field has a serious effect on the crystal structure and polarization properties of BTO. Under the applied electric field, Ti ions shift from a random position to aligned positions, obtaining high polarization and a high dielectric constant [61]. To analyze the effect of an electric field, we applied a 1 kVcm^−1^ electric field at a higher temperature (above *T*_B_), and then, the Brillouin spectra were taken in the field cooling process. A significant difference was noticed between $\vartheta_{LA}$ and $\Gamma_{LA}$ in both the cubic and tetragonal phases. It has already been discussed and reported that in BTO crystals, the Ti ions fluctuate along eight equivalent [111] directions inside a PNR [53]. Owing to the application of an electric field along the [100] direction, the density of dynamic/static PNRs can be decreased, which is expected to weaken the LA phonons’ scattering by PNRs, resulting in a higher frequency shift and lowering the damping of the LA phonons. However, it is significant to note that *T*_B_ is not influenced by the applied electric field, and T* and *T*_c_ change after applying electric field. This observation is in good agreement with acoustic emission results [58]. According to acoustic emission results, the electric field dependence is stronger in T* than *T*_c_ due to the possible field ordering of PNR dipoles, which could favor their freezing. In the tetragonal phase, a significant change between $\vartheta_{LA}$ and $\Gamma_{LA}$ has been observed. Due to the sufficiently strong electric field, the correlation among the PNRs becomes sluggish, and the alignment of nanodomains/static PNRs is facilitated and enables complete switching into the macro/single-domain state at *T*_c_, giving rise to the formation of the ferroelectric state. In addition, the large differences in $\vartheta_{LA}$ and $\Gamma_{LA}$ between ZFC and FC indicate the transformation from the ferroelectric [100] direction to the [001] direction in the tetragonal phase. Upon the subsequent heating (heating after field cooling), the crystals transform back to their initial state, which results in hysteresis below *T*_c_.

**Figure 5:**
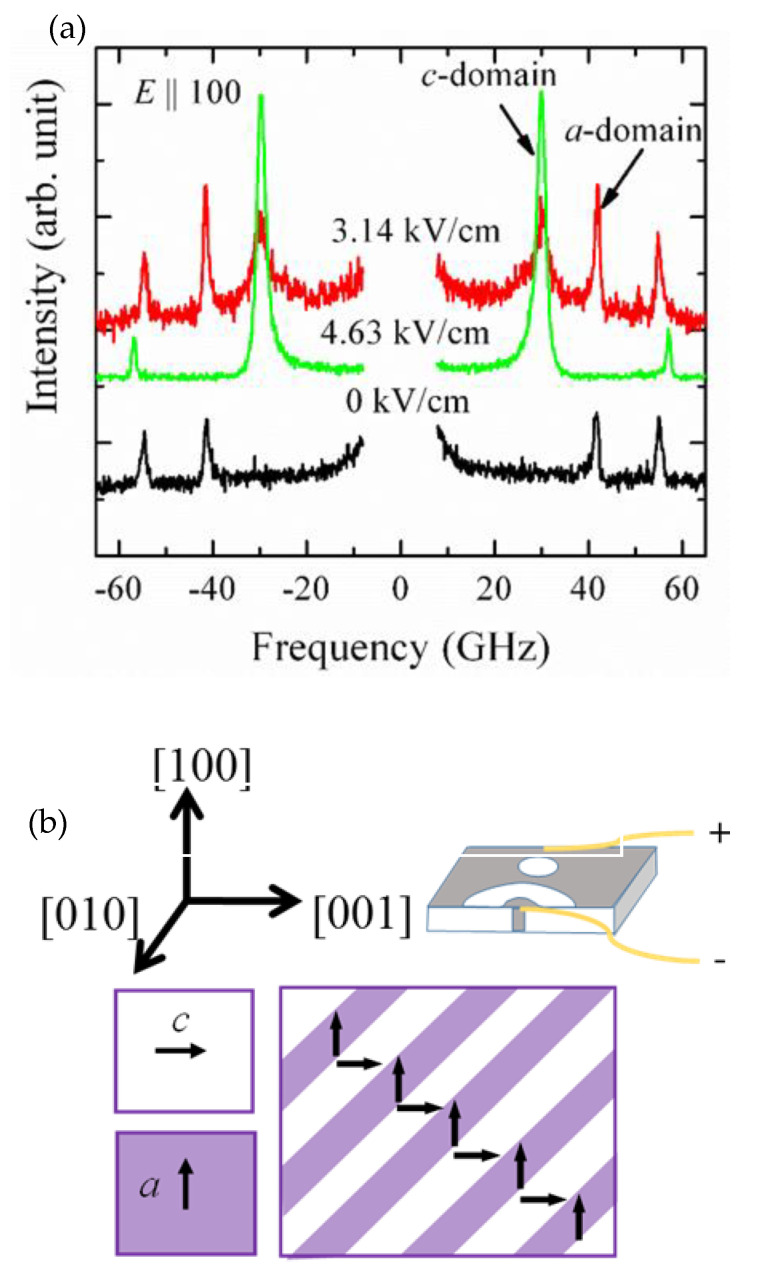
(**a**) A set of typical Brillouin scattering spectra BTO single crystals in the ferroelectric tetragonal phase at some selected electric fields at 303 K; (**b**) shows the orientation of a and c domains.

In ferroelectrics, to understand the domain switching process, many research works have been performed and remarkable progress has been achieved [62,63,64,65,66]. Previously, it was reported that under an applied electric field antiparallel to the polar direction, only 180° domain switching can be possible in ferroelectrics. However, non-180° domain switching was also suggested by both the theoretical and experimental observations [67,68]. Under the application of an external electric field, now, it has been established that two types of domains can nucleate: 90° domains with polarization perpendicular to each other. which minimizes strain via the formation of twin walls, and 180° domains having anti-parallel spontaneous polarization, which diminishes the depolarization fields [69]. To observe the domain switching effect, a set of typical Brillouin scattering spectra in the tetragonal phase at 303 K is shown in Figure 5.

When there is no electric field, the transverse acoustic (TA) mode is denoted as the *a*-domain, since the sample is cut along the [100]-oriented plate. When the field is increased up to 2.94 kV/cm, another TA mode at around 30 GHz begins to appear and persists up to 3.9 kV/cm. The additional TA mode in BTO crystals means the appearance of the *c*-domain. Within 2.94 and 3.9 kV/cm, both domains co-exist. Above 3.9 kV/cm, the intensity of the additional TA mode goes to the maximum and saturates, suggesting that the 90° domain switching has completed. The 90° domain switching was also noticed by Li et al. [70] and Salje et al. [58]. Moreover, whether the enhanced polar properties are related to the engineered domain structures or whether the increased density of the domain boundaries between domains plays an important role in BTO crystals is an open question.

### 3.2. Local Lattice Disorder in BaZrO_3_ Single Crystals

**Figure 6:**
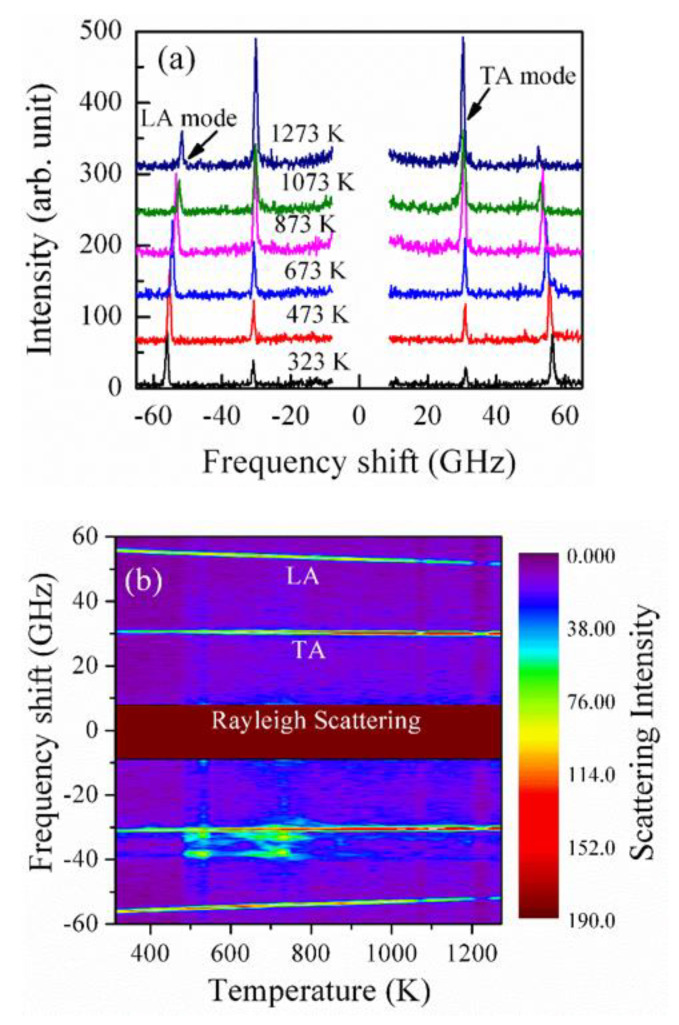
(**a**) A set of typical Brillouin scattering spectra at some selected temperatures and (**b**) a contour color map of intensity versus temperature and frequency of BZO single crystals [13].

The perovskite structure of BZO single crystals, long known to maintain their cubic symmetry at room temperature without any structural phase transition with wide temperature variations, has recently been exhibited to have various ground state structures with low symmetries involving octahedral rotation. However, the ground state structure in BZO is questionable. Several first-principles calculations reported that the true structural state of BZO may be tetragonal *I4/mcm* because of the unstable antiphase antiferrodistortive phonons of oxygen octahedra [69,71]. These local tetragonal distortions can be averaged out and may not be possible to detect using X-ray or neutron diffraction experiments, which results in the cubic symmetry. From the quantum mechanical viewpoint, Brillouin scattering is assumed as one of the interactions of light photons with acoustic or vibrational quanta such as phonons. No coupling between the ultrasonic transducer and other devices with samples is needed to produce acoustic excitation and detection in Brillouin scattering, which leads to accurately measuring the acoustic properties. Figure 6a shows a set of Brillouin scattering spectra at some selected temperatures, and a contour color map is displayed in Figure 6b.

**Figure 7:**
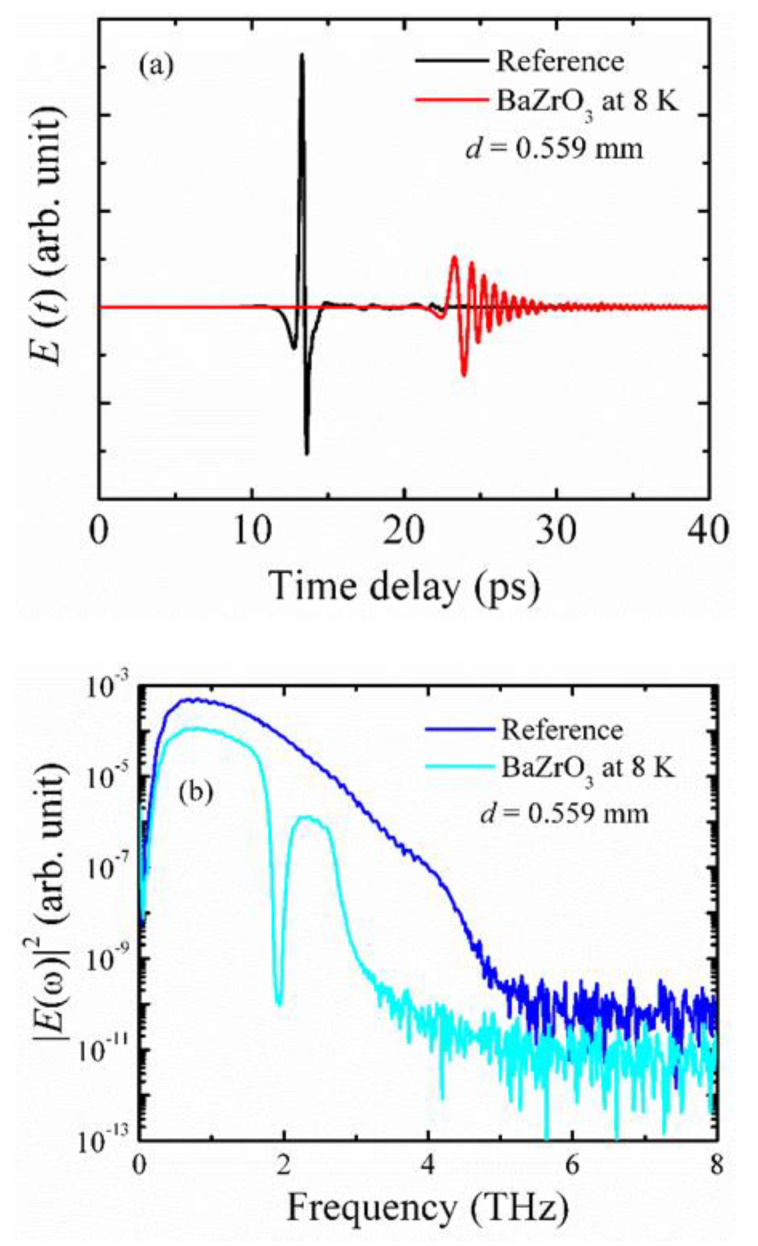
(**a**) Time-domain waveform and (**b**) the power spectra of a BaZrO_3_ single crystal converted from time-domain waveforms at 8 K.

We observed clearly an LA and a TA phonon mode. From the temperature dependence of these phonon modes, no sharp discontinuity related to a phase transition was observed. Therefore, macroscopically, it remains as the ground state structure at room temperature. However, the most striking feature of this Brillouin spectrum is the appearance of the TA mode, which exists up to 1273 K. The Brillouin spectra were recorded in a backward scattering geometry, and only the LA mode should propagate along the [100] direction based on the selection rules in the cubic symmetry [72]. Due to this, several previous experiments noticed only the LA phonon mode in the cubic symmetry [73]. Therefore, the strong TA mode, not allowed in the cubic symmetry, reflects the presence of local lattice distortion, which can destroy the center of the symmetry in a cubic matrix. This local lattice distortion is also evident from terahertz time-domain spectra (THz-TDS). Figure 7a,b shows the time-domain waveform and transmission power spectra converted from the time-domain waveform of a BZO single crystal at 8 K, where a clear absorption peak at about 1.94 THz is observed.

In cubic perovskite with the space group *Pm*$\bar{3}$*m*, three IR active transverse optic (TO) modes are allowed according to the selection rule. Two former measurements on the IR phonon spectra have been reported [74,75]. In addition, the IR phonon frequencies have been reported by first-principles calculations [76,77]. In comparison with those published in BZO ceramics by IR and first-principles calculations, the observed TO mode was observed in THz-TDS at a very low frequency, which reflects the additional new mode in a BZO crystal. The FTIR spectra of BZO ceramics [74] show three narrow phonon peaks, as expected for a simple cubic perovskite structure. Among three phonon modes, the TO2 mode was the strongest one and was assigned as the Slater mode (Zr-O_6_ stretching vibrations). The eigenvector of the lowest-frequency TO1 mode was close to the last mode, playing the role od the ferroelectric soft mode. The highest-frequency mode above 15 THz was due to the O_6_ deformation and called the Axe mode. In addition, to fit the FTIR spectra, Nuzhnyy et al. [74] used the nine oscillator models and concluded that not all the oscillators have a clear meaning. However, the appearance of a new mode at 2 THz in a BZO crystal and an additional mode at 9 THz in BZO ceramics [74] indicated that BZO has a lower symmetry than cubic Pm*Pm*$\bar{3}$*m* and/or these modes come from the local lattice distorted structure. Very recently, D.H. Gim et al. measured the Raman and IR spectra of both the ceramics and single crystals of BZO [75]. They also observed the additional phonon modes in both samples and reported that these additional phonon modes are due to the presence of local distortion or disorder. Therefore, the additional phonon modes in Brillouin scattering, THz-TDS, Raman spectra [75], as well as IR spectra [74] can be considered evidence of the presence of the local lattice distorted structure in BZO. 

### 3.3. Lattice Instability and IR Spectra of BaZrO_3_ Crystals

Tolerance factor *t* is a well-established parameter, which is used to identify the stable structure. The formula for *t* of ABO_3_-type perovskite is given by

(3) $$t=\frac{R_{A-O}}{R_{B-O}\sqrt{2}}$$

**Figure 8:**
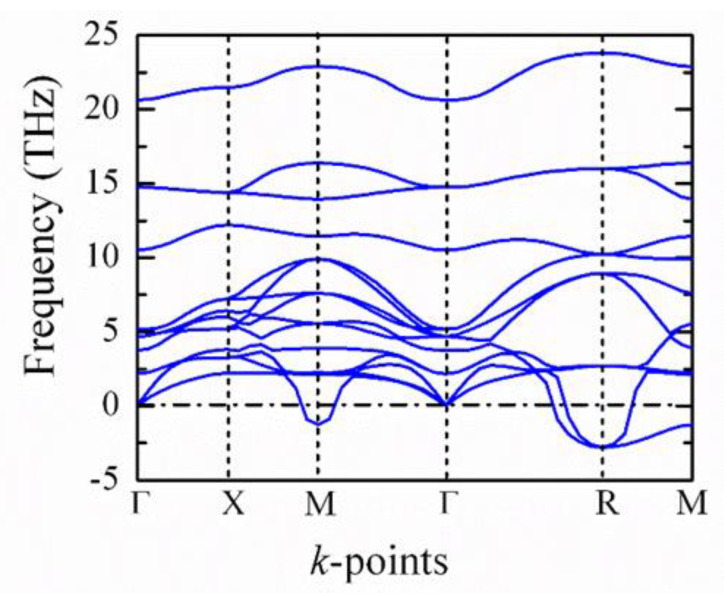
Phonon dispersion relation of BZO crystals [13].

Here, $R_{A-O}$ is the sum of A and O ionic radii and $R_{B-O}$ is the sum of B and O ionic radii. Generally, t<1 leads to the rotation and expansion of B-O_6_ octahedra. A low-temperature anti-ferroelectric phase is generated due to the octahedral rotations. If t>1, the B-O_6_ octahedra are stretched from their preferred B-O bond lengths, resulting in the distortions of the B cation by creating room for the B cations to move to the off-center position. In BZO crystals, the values of the ionic radii of Ba and Zr atoms are 1.61 Å and 0.72 Å, respectively, which leads to t=1, so the cubic structure should have no driving force to deform to a lower symmetry. However, the calculation of the phonon spectrum of BZO using DFT calculations is unfavorable to achieve the stable structure of BZO. The phonon dispersion relation for the five-atom unit cell of BZO is shown in Figure 8. The very important characteristics of the phonon spectra are related to the lowest-frequency acoustic phonon branch, which goes to negative at the R- and M-points in the Brillouin zone. However, theses unstable phonon modes were absent in. [78,79], but appeared in [69], where all the calculations were performed using the DFT method. The negative phonon frequencies correspond to the imaginary frequencies, which implies the change of the crystal symmetry. Due to the anharmonic phonon interaction, the negative phonon frequencies may also appear, which are responsible for a structural phase transition of BZO with respect to some external conditions. Therefore, BZO is dynamically unstable, which can change in phase under pressure or an electric field. This observation is consistent with the present Brillouin scattering and THz-TDS results. Indeed, under the applied pressure, very recently, the cubic to rhombohedral *R*$\bar{3}$*c* and rhombohedral to tetragonal *I4/mcm* phase transitions at 8.4 and 19.2 GPa, respectively, were observed [75]. A few years before, Yang et al. also observed the pressure dependence of the phase transition from the cubic to tetragonal *I4/mcm* at 17.2 GPa [70]. We also observed the pressure dependence of the phase transition by first-principles calculations [13].

**Figure 9:**
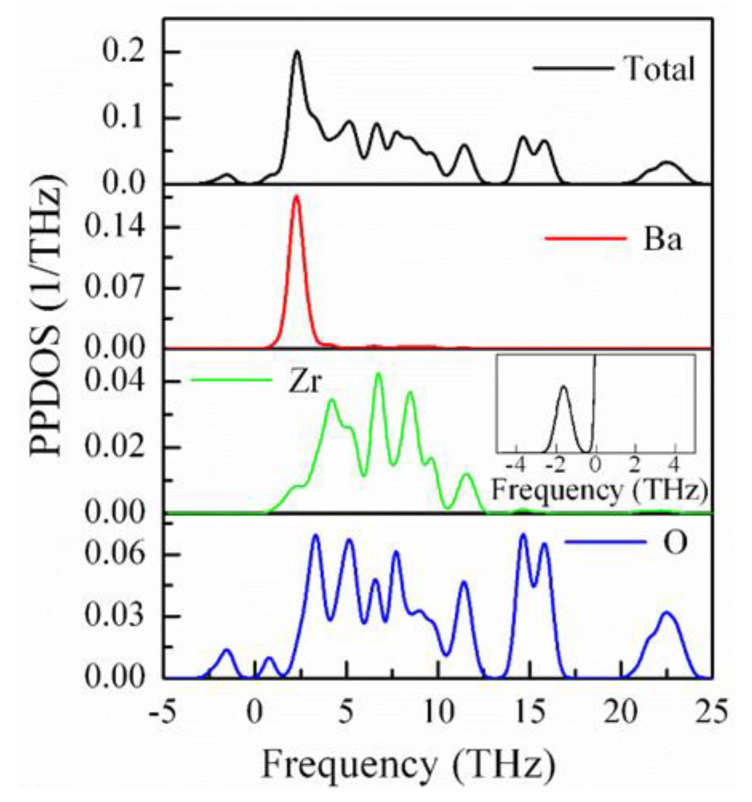
Partial and total phonon density of states of BZO crystals. Inset shows the contribution of Zr atom.

The atomic contribution in the phonon spectra can be seen from the partial phonon density of states (PPDOS), which is shown in Figure 9. It can be noticed from the PPDOS that the negative phonon frequency appears due to the vibration of Zn and O atoms. Therefore, it can be concluded that within the frequency range from 0.5 to 3.0 THz, an unstable phonon mode at the *R*- and/or *M*-points of the Brillouin zone is responsible for the phase transition and the superior dielectric and piezoelectric responses of BZO, which correspond to the tilts of the ZrO_6_ octahedra. Among the perovskite ferroelectric oxides such as PZT [80], this type of unstable phonon mode is observed commonly at the *R*- or *M*-points of the Brillouin zone.

**Figure 10:**
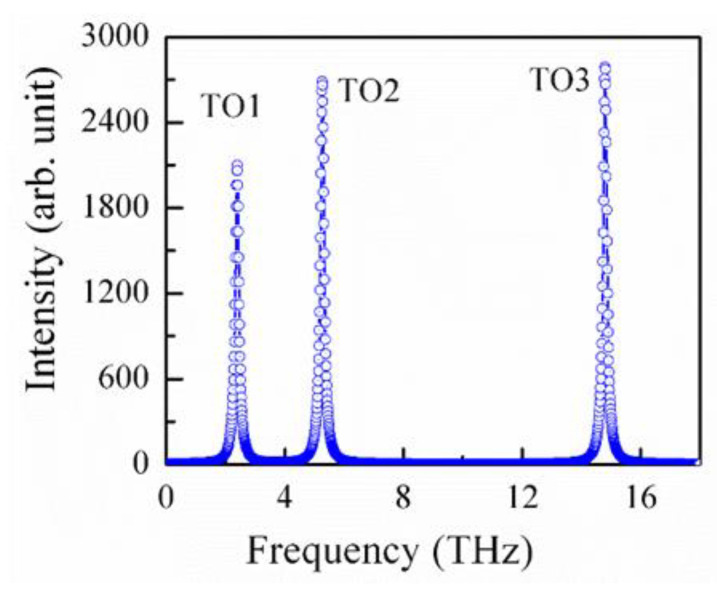
Calculated IR active TO mode frequencies of BZO crystals in the cubic phase.

Finally, we also estimated the IR spectra of BZO using first-principles calculations. According to the group theoretical analysis, due to the cubic symmetry, three phonon branches of the T_1u_ symmetry is IR active, and one branch of the T_2u_ symmetry is not IR, nor Raman active. The estimated IR spectra of the BZO single crystal are displayed in Figure 10 where the frequencies of the three TO modes are 2.4, 5.3, and 14.8 THz, respectively.

These values of IR active modes are in good agreement with previous theoretical results [72,76]. However, Nuzhnyy et al. [74] obtained the values of these IR active modes to be 3.48, 6.4, and 15.6 THz, respectively, which are higher than the present calculated values. It is well known that the mode frequencies obtained from DFT calculations may vary due to the choice of different functionals and pseudopotentials, as shown for BZO, as well as for BTO. The origin of the additional modes observed by Nuzhnyy et al. and our THz-TDS has not been discussed anywhere using first-principles calculations. J. W. Bennett et al. estimated the values of IR modes by considering the *P*$\bar{1}$ symmetry [71]. However, their reported values also cannot explain the experimentally observed additional modes in BZO. Therefore, further theoretical calculations are not only necessary, but will also be interesting.

### 3.4. First-Principles Calculations of BaCuO_3_ Crystals

**Figure 11:**
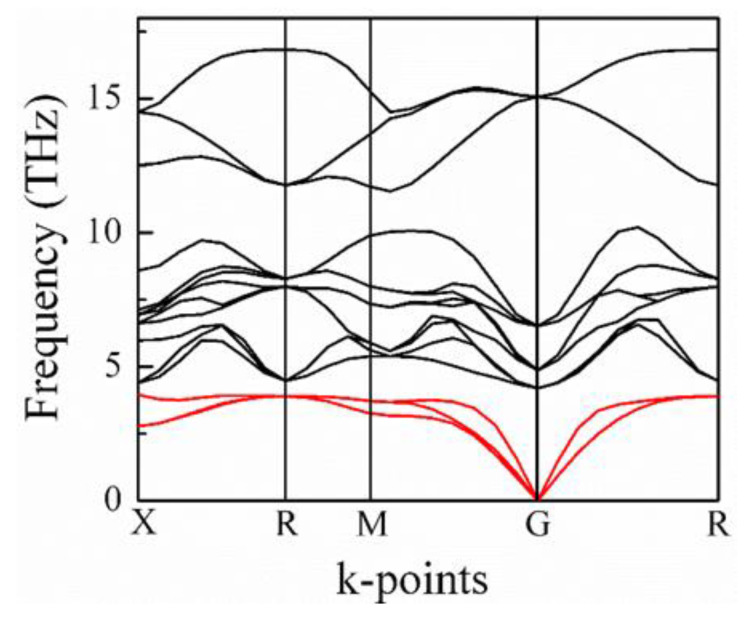
Phonon spectra of BaCuO_3_ at zero pressure.

**Figure 12:**
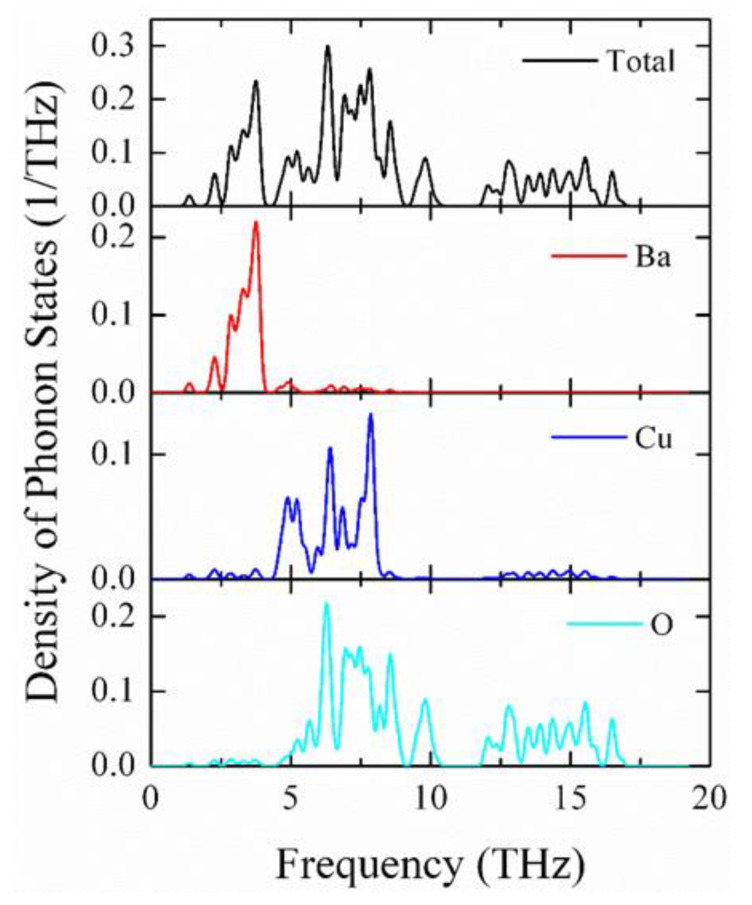
Density of phonon states of BaCuO_3_ at zero pressure.

Very recently, another important type of perovskite, BCO, was discovered [36]. BCO belongs to the family of chemically simple perovskite oxides. At a fundamental level, to know the dynamical stability of BCO over the whole Brillouin zone boundary, the phonon dispersion relation is very important. As discussed above, the observation of negative phonon frequencies corresponds to the instability of the crystal symmetry; otherwise, they are considered as dynamically stable. The calculated phonon spectra and PPDOS of BCO at absolute zero temperature along the principal symmetry direction of the Brillouin zone are shown in Figure 11 and Figure 12, respectively. As for BZO, negative phonon frequencies are absent in BCO. On the other hand, at the *G*-point, the positive value of the low-frequency acoustic phonon (indicated by red color) clearly reflects that BCO single crystals are dynamically stable. So far, there have been no available data, neither experimental, nor theoretical, to compare the present results. From the PPDOS, it is seen that the low-frequency region (1–4 THz) is mainly due to the vibration of Ba atoms, while the intermediate-frequency region (4–8 THz) arises because of the vibration of both the Cu and O atoms. The high-frequency region (above 8 THz) is composed of the vibration of O atoms due to the lower mass.

The elastic properties of a material are essential to study because of its response under different external loads. this also helps predict the numerous applications of a solid in different sectors by exploring the outstanding physical properties, such as elastic stiffness, bulk, modulus, compressibility, Young’s modulus, ductility, etc. The independent elastic constants for cubic symmetry, namely *C*_11_, *C*_12_, and *C*_44_, of BCO have been calculated using the stress–strain method [81,82]. All the estimated elastic constants are positive and satisfy the mechanical stability criteria $(C_{11}-C_{12})>0,\;\left(C_{11}+2C_{12}\right)>0,\;\mathrm{and}\;C_{44}>0$. Therefore, BCO crystals are stable from the viewpoint of both mechanics and thermodynamics.

**Figure 13:**
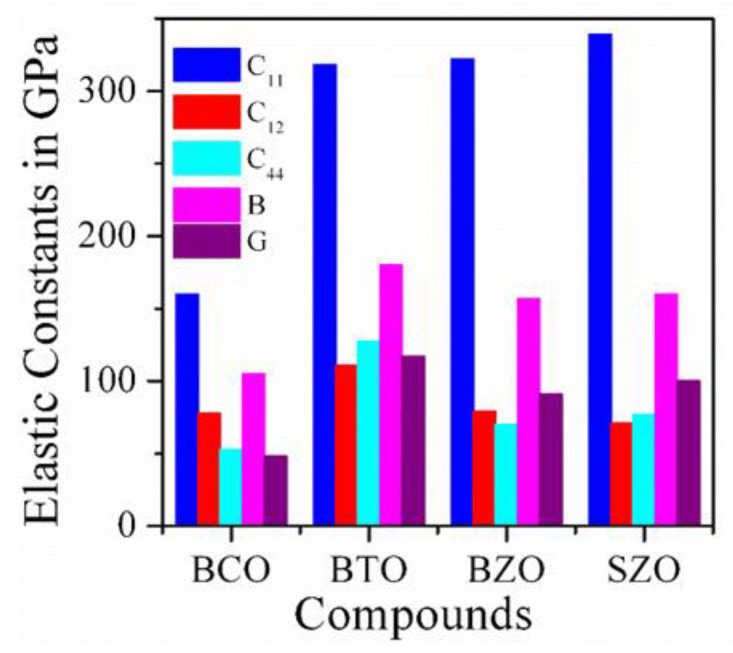
Comparison of different elastic moduli of BaCuO_3_ with other perovskites.

For the material containing the cubic phase, the equations for the calculation of *B* and *G* are

(4) $$B_{V}=B_{R}=\frac{1}{3}{(C}_{11}+{2C}_{12})$$

(5) $$G_{V}=\frac{1}{5}(C_{11}-C_{12}+{3C}_{44})$$

(6) $$G_{R}=\frac{{5(C}_{11}-C_{12})C_{44}}{C_{44}+{3(C}_{11}-C_{12})}$$

(7) $$B=\frac{B_{V}+B_{R}}{2}$$

(8) $$G=\frac{G_{V}+G_{R}}{2}$$

where *B_V_* and *G_V_* represent the Voigt approximation, *B_R_* and *G_R_* indicate the Reuss approximation, and *B* and *G* have been calculated following the Voigt–Reuss–Hill approximation. The hardness of a material strongly depends on *C*_44_ and *G*. In order to check the hardness of the other well-known perovskites, we compared our results with BTO, BZO and SrZrO_3_, which is shown in Figure 12. From Figure 13, it can be noticed that BCO has a smaller value of not only *B* and *C*_44_, but also the other elastic moduli, which should result in BCO being a soft material. Therefore, in comparison with those perovskites, BCO is more suitable for use as a thermal barrier coating substance.

The most interesting phenomena observed in BCO is that it exhibits superconductivity at low temperatures. Recently, for Ba_2_CuO_4-y_, *T*_c_ was found to be around 73 K [83]. We also estimated *T*_c_ by using the Allen-Dynes modified McMillan equation [84] as

(9) $$k_{B}T_{c}=\frac{\hbar \omega_{log}}{1.2}\exp\left[-\frac{1.04\left(1+\lambda \right)}{\lambda -\mu^{*}\left(1+0.62\lambda \right)}\right]$$

where λ is the electron–phonon coupling constant, defined as $\lambda =N\left(E_{F}\right)V_{e-ph}$, with $V_{e-ph}$ denoting the electron–phonon interaction energy responsible for the Fermi surface instability and Cooper pairing, and $\mu^{*}$ is the repulsive Coulomb pseudopotential. The values of $\omega_{log}$, λ, and $\mu^{*}$ were obtained as 121, 0.84, and 0.01, respectively, using the calculation by the quantum ESPRESSO code. These values were used to calculate *T*_c_ using the above equation, and we obtained *T*_c_ = 9.96 K. The large difference arose because of the oxygen defect. It has already been mentioned that the oxygen defect can increase the *T*_c_ of a material [85,86]. However, here, we report the *T*_c_ for the pure BCO structure.

**Figure 14:**
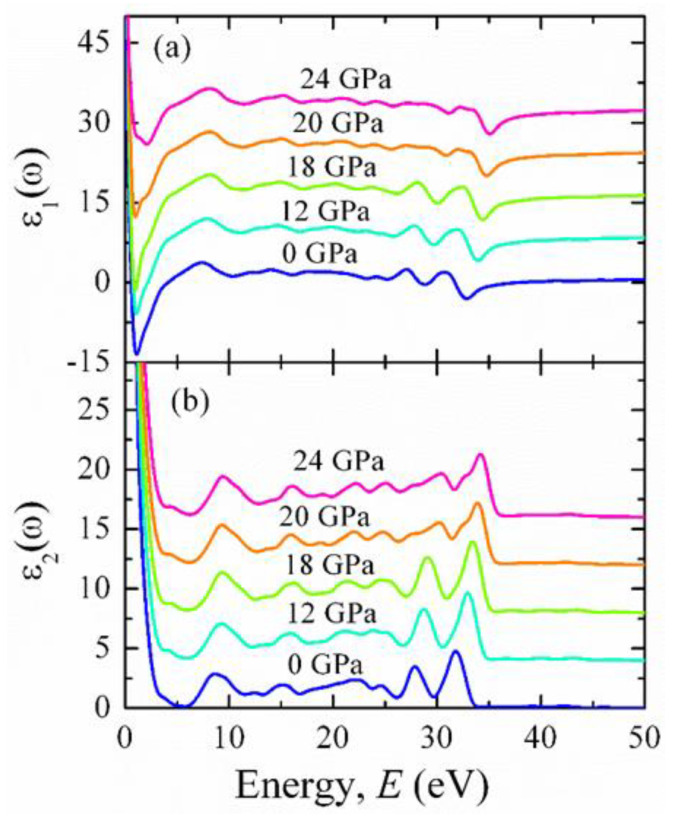
The energy dependence of the (**a**) real and (**b**) imaginary part of the dielectric constant of BaCuO_3_ at some selected pressures. Different offset values are set to visualize the optical spectra clearly.

At some selected pressures, the real (ε_1_(ω)) and imaginary (ε_2_(ω)) parts of the dielectric constants are displayed in Figure 14a,b, respectively. The low-energy optical response is dominated by ε_2_(ω) for metallic compounds. From Figure 14a, it is seen that ε_1_(ω) crosses zero from the bottom level at 37.9 eV, and at the same energy, ε_2_(ω) approaches zero, which reflects the metallic nature of BCO. It also suggests that the material becomes transparent when the energy of the incident photon exceeds 37.9 eV. Due to the intraband transition, the prominent peaks are observed in both ε_1_(ω) and ε_2_(ω) at around 27 and 31 eV. The peak at around 27 eV almost diminishes, when the applied pressure is 18 GPa.

**Figure 15:**
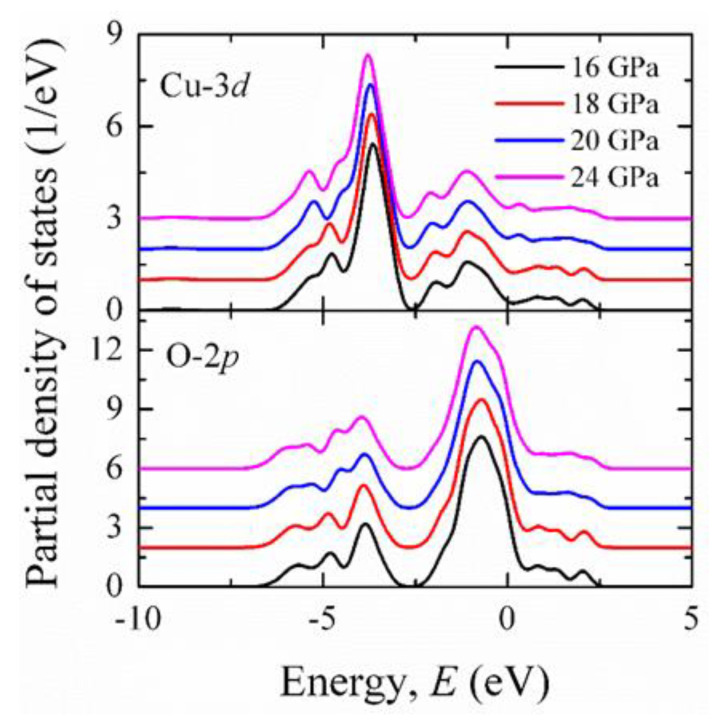
Partial DOS of Cu-3d and O-2p orbitals at 16, 18, 20, and 24 GPa.

To explain these observations, we calculated the pressure dependence of the partial density of electronic states, which is shown in Figure 15. It can be noticed from the pressure dependence of the partial density of states that there is a prominent hybridization between the neighboring Cu-*d*_*x*^2^−*y*^2^_ and O-2*p_x_* orbitals at 18 GPa, which is supposed to screen the Cu core holes, resulting in the decrease of the peak intensity.

**Figure 16:**
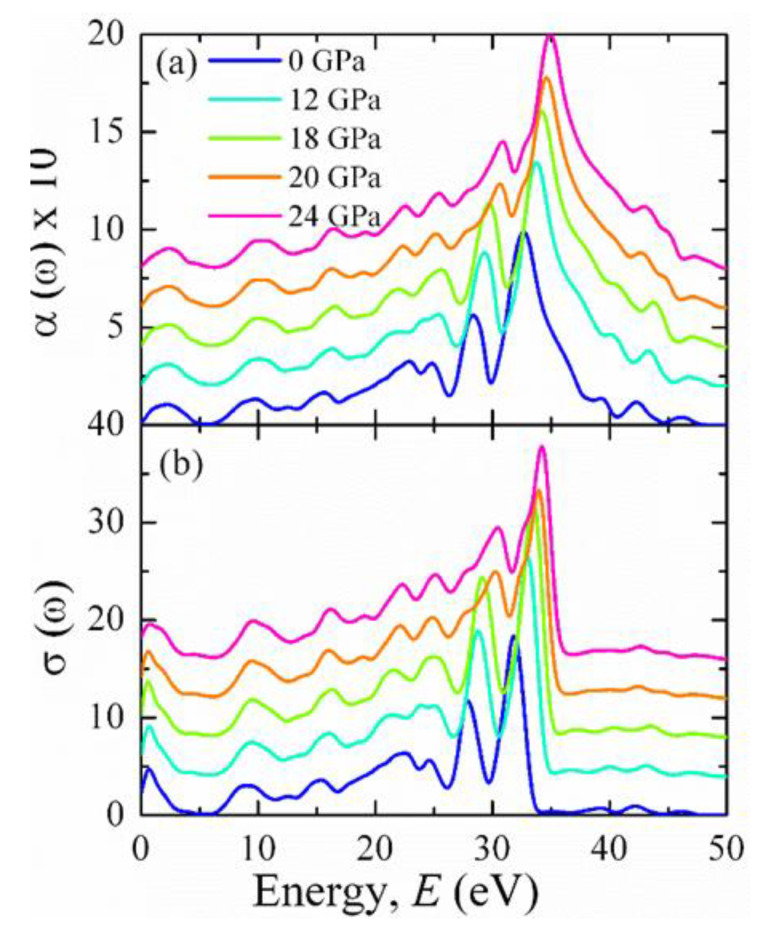
The energy dependence of (**a**) absorption coefficient and (**b**) conductivity of BaCuO_3_ at some selected pressures. Different offset values are set to visualize the optical spectra.

Significant absorption at zero photon energy was observed in BCO, as shown in Figure 16a, indicating the metallic nature. Many peaks were observed in the absorption spectrum. However, the intensities of these peaks in the IR and visible regions were small compared to the UV region. At around 27 and 31 eV, two intense peaks were observed. As a result, the solar spectrum in UV regions can be absorbed by BCO. The real part of the photoconductivity (σ) of the BCO crystal is displayed in Figure 16b. It is seen that σ has a finite value at zero photon energy, which reflects the metallic conductivity of BCO, totally consistent with the results of the absorption and dielectric constant. The finite value of σ can be increased owing to the absorption of the photon with sufficient energy. The maximum value of σ was obtained in the UV region. A sudden jump and decrease of intensity of a peak around 27 eV can be noticed from Figure 16a,b with increasing pressure, which might be associated with the transition from the initial state 2*p*^6^3*d*^9^ to the final state 2*p*^5^3*d*^10^. Overall, there are no studies on pure BCO to compare the result. Many studies are required for the superconducting BCO to understand the basic mechanism of its superconductivity.

## 4. Summary

The crystal structure of perovskite oxide has a variety of compositions and component elements, which give rise to a wide range of applications due to its unique properties. Here, three different types of perovskites were reviewed by means of Brillouin scattering and first-principles studies.

In BTO single crystals, a significant softening of the frequency shift and FWHM of the LA phonon is observed in the vicinity of a cubic–tetragonal phase transition temperature. The Burns temperature, *T*_B_ = 533 K, and intermediate temperature, T* = 503 K, were identified, which show very close agreement with recent acoustic emission measurement result. When the sample was cooled down with an electric field, an increase of the frequency shift and a decrease of the FWHM in both the cubic and tetragonal phase were observed due to the reduced density of the twin domain walls and static/dynamic PNRs. A complete 90° domain switching process was noticed under the application of an electric filed in the ferroelectric tetragonal phase. In the BZO single crystal, no phase transition was observed from 8 K to 1273 K, reflecting that the BZO system is macroscopically cubic. The presence of a TA phonon in the Brillouin spectra suggests local lattice distortion, which destroys the center of symmetry in the cubic matrix. This observation is also justified with the observation of an additional absorption peak near 2 THz in the THz-TDS experiment. The imaginary part of the frequency in the phonon dispersion spectra suggests structural instability with respect to oxygen octahedral rotations. In BCO, the dynamical and mechanical stabilities were confirmed from the calculation of the phonon spectra and elastic constants using first-principles calculations. The magnitude of different elastic constants of BCO compared with other related perovskites shows that BCO is a mechanically stable soft perovskite with improved ductility. The superconducting transition temperature, *T*_c_ = 9.96 K, was obtained from phonon calculations. The change of the peak position and the reduction of the peak intensity in various optical constants with pressure were in good agreement with the observation of a pressure-dependent partial DOS. The shift of the peak position at 18 GPa in different optical parameters was attributed to the transition from the initial state 2*p*^6^3*d*^9^ to the final state 2*p*^5^3*d*^10^ of the Cu atom. Since there are no experimental reports on cubic BCO available, the results discussed here will be very helpful, not only for future experimental and theoretical research, but also for the assessment of the suitability of BCO in different optoelectronic devices.

## Data Availability

Not applicable.
